# Factors influencing women’s decisions to drink alcohol during pregnancy: findings of a qualitative study with implications for health communication

**DOI:** 10.1186/1471-2393-14-246

**Published:** 2014-07-24

**Authors:** Carla S Meurk, Alex Broom, Jon Adams, Wayne Hall, Jayne Lucke

**Affiliations:** Postdoctoral Research Fellow, The University of Queensland, UQ Centre for Clinical Research, Royal Brisbane and Women’s Hospital Site, Herston, Queensland 4029 Australia; Sociology, Australian Research Council Future Fellow, The University of Queensland, School of Social Science, St Lucia, Queensland 4072 Australia; Public Health, National Health and Medical Research Council Career Development Fellow, University of Technology Sydney, Faculty of Health, PO Box 123, Broadway, NSW 2007 Australia; Australia Fellow, The University of Queensland, UQ Centre for Clinical Research, Royal Brisbane and Women’s Hospital Site, Herston, 4029 Queensland; Principal Research Fellow, The University of Queensland, UQ Centre for Clinical Research, Royal Brisbane and Women’s Hospital Site, Herston, 4029 Queensland

**Keywords:** Pregnancy, Alcohol, Qualitative research, Risk, Consumption, Patient-practitioner communication, Public health promotion

## Abstract

**Background:**

Despite Australian guidelines advising abstinence from alcohol during pregnancy, a relatively high number of Australian women continue to drink alcohol while pregnant. While some call for greater advocacy of the need for abstinence, others have expressed concern that abstinence messages may be harmful to pregnant women and their unborn babies due to the anxiety they could provoke. We present findings on women's deliberations over drinking alcohol during pregnancy, particularly their emotional dimensions, to inform debates about public health messages and practitioner-patient discussions regarding alcohol use during pregnancy.

**Methods:**

Semi-structured face-to-face interviews were conducted with 40 women in their homes. Our sample comprised women aged 34–39, drawn from the Australian Longitudinal Study on Women’s Health, living in the Greater Brisbane Area who were pregnant, or had recently given birth, in 2009. An inductive qualitative framework analysis approach was used to identify and interpret themes explaining why pregnant women choose to drink or not.

**Results:**

Women generally described drinking small amounts of alcohol during pregnancy as being a low risk activity and talked about the importance of alcohol to their social lives as a reason for continuing to drink or finding abstinence a burden; sensitisation to the judgements of others was not widespread. Women predominantly assessed the risk of their drinking in terms of the kinds of alcoholic beverages consumed rather than alcohol content. In reflecting on the advice they recalled receiving, women described their healthcare practitioners as being relaxed about the risks of alcohol consumption.

**Conclusions:**

The significance of alcohol to women’s identity appeared to be an important reason for continued alcohol use during pregnancy among otherwise risk averse women. Anxiety about alcohol consumption during pregnancy was not widespread. However, obstetricians were an important mediator of this. Health messages that dispel the notion that wine is a “healthy” choice of alcoholic beverage, that provide women with strategies to help them avoid drinking, that advise the broader public not to pressure women to drink if they do not want to, and educate women about the effects of ethanol on maternal and fetal bodies, should be considered.

## Background

Since 2009, Australian government guidelines have stated that: “for women who are pregnant or planning a pregnancy, not drinking is the safest option” [1]. Yet, rates of alcohol consumption by Australian women during pregnancy are among the highest in OECD countries. Data collected from the Australian Longitudinal Study on Women’s Health (ALSWH) show that approximately 80% of Australian women consumed alcohol during pregnancy over the period 1996–2006 [2], a rate that dropped to 72% in 2009 [3]. Results from the 2010 National Drug Strategy Household Survey suggest lower rates of 47.3% of women consuming alcohol during pregnancy prior to pregnancy recognition and only 19.5% consuming alcohol after recognising that they were pregnant [4]. However, even these latter figures are high compared to rates of maternal drinking during pregnancy of 12% among Swedish women [5] and around 10% in the USA [6]. The relatively high proportion of Australian women who drink during pregnancy is at odds with women’s normative beliefs about whether pregnant women should drink alcohol. Studies show that over three quarters of Australian women think that women should not drink alcohol during pregnancy and that they hold a negative view of pregnant women who do [7].

Studies from Australia and abroad show that preconception alcohol intake consistently predicts maternal alcohol consumption [2, 3, 8–10]; it may be that patterns of drinking, rather than rates of alcohol consumed, are important predictors [8]. Despite numerous quantitative studies on alcohol consumption, there are surprisingly few qualitative studies that have explored this topic. An exception, a study of 12 Australian women and 12 midwives investigating why women chose *not* to drink during pregnancy, found that they chose to abstain during pregnancy due to a “sense that [alcohol] was generically harmful” and because drinking would make them feel guilty about breaking a perceived social norm against drinking while pregnant [11], p71. However, these women also described how “the social expectation of drinking alcohol” in Australian society made it difficult to remain abstinent, particularly before they told others that they were pregnant [11], p71.

Such evidence points to the contradictory social norms pregnant women must navigate, a matter which deserves considered attention. We need to explore the possible impacts of such contradictory messages on women as it guides both their decisions to drink or abstain, their sense of wellbeing during pregnancy and the strategies we employ to help women make informed choices. The lack of evidence as to whether low-moderate alcohol intake during pregnancy poses risk to the fetus [12] maybe part of what is fuelling this cultural double bind. Adding to this, some commentators have voiced concern that the risk of advocating abstinence might pose a risk to the fetus inasmuch as it could provoke anxiety among women who drink while pregnant [13]. Some argue that imposing a norm of abstinence for pregnant women constitutes an unwarranted form of “coercive control” over women’s bodies [14]. There have been concerns raised that abstinence messages may cause anxiety in women who unknowingly consume alcohol before discovering they are pregnant and that, in the worst case, such anxiety may lead some women to consider abortion [15]. The published evidence does not support the latter as a likely outcome [16]. However, much of this debate has taken place in philosophical writing and opinion based editorials, without supporting evidence. Despite this lack of evidence, these concerns have been picked up and reported in mainstream media [17].

Qualitative studies have investigated the role of midwives as communicators of information about alcohol consumption, finding that both women and midwives were comfortable talking about alcohol consumption but such conversations did not routinely take place [18, 19]. However, there has been no research that expressly investigates the emotional dimensions of women’s experiences of receiving information about abstaining from alcohol during pregnancy, whether from healthcare professionals or through other social or media networks. There is a similar lack of research presenting the perspectives of women who choose *to* drink during pregnancy. These are important gaps, especially given a publicised expression of concern about the possibility of causing anxiety among expectant mothers who consume alcohol.

This study aims to inform debates about strategies for discussing alcohol consumption with pregnant women in ways that are effective but sensitive to preserving women's emotional wellbeing. We report findings from a study on Australian women's experiences and decision making during pregnancy, labour and birthing. The study focussed specifically on the sources of knowledge and expertise women drew on at this time. The paper examines how women contextualised and acted upon their risk perceptions, the influence of personal identity on alcohol consumption, lay concepts of ‘drinking guidelines’, and women’s recollections of discussing alcohol use with their practitioners.

## Methods

### Sample characteristics

Our sample comprised 40 women from the 1973–78 cohort of ALSWH who were resident in the Greater Brisbane Area of Australia. ALSWH is a study of over 40,000 Australian women that began in 1996. It comprises women in three age groups, randomly selected from the national Medicare database and invited by mail to participate. Women in the “younger” cohort, aged between 18 and 23 when the study began in 1996 (34–39 in 2012), who were pregnant or had recently given birth when surveyed in 2009, were invited to participate in our broader study of women’s experiences and decision-making during pregnancy, labour and birthing. 75 women in the Greater Brisbane Area satisfied these criteria and agreed to participate. A list of these women was generated and every second woman was telephoned and invited to take part in a qualitative interview with CM. We returned to the beginning of the list to resample until the quota of 40 interviews was filled. This sample size proved sufficient to achieve thematic saturation [20]. Our sample predominantly comprised White Australian women and was skewed towards those of higher socioeconomic status, with 68% (n = 27) having used private healthcare (including consulting an obstetrician throughout pregnancy) during pregnancy, labour, and birth. Previous studies have indicated that socioeconomic status and age are positively associated with alcohol consumption during pregnancy [4, 21] so it is particularly important to examine the views of this group of women. Approximately 9% of women contacted (n = 4) refused to participate or were unable to participate.

### Data collection techniques

Following ethical clearance by the Human Research Ethics Committee, University of Newcastle, the Human Research Ethics Committee, University of Technology Sydney and the Human Research Ethics Committee, University of Queensland, CM completed face-to-face semi-structured interviews with women at their homes during April and May 2012. Women provided written consent to participate prior to the interview commencing. Interviews lasted between 30 minutes and one hour and were transcribed verbatim. The aim of the study was to elicit women’s health care experiences and decision making during pregnancy, labour and birthing with a focus on the sources of knowledge and expertise women consulted. As part of this study, women were asked “during your pregnancy, were you particularly conscious of the foods you ate or the drinks you consumed?” This broad question opened up a free-flowing conversation with women about their specific consumption practices. A similarly broad question “what advice did you receive from your doctor/obstetrician?” elicited responses on a range of issues that CM explored systematically with the interviewee. Experiences and decision-making about alcohol use emerged spontaneously as a prominent theme in women’s discussions of these topics. In other words, CM neither encouraged nor discouraged women to discuss their alcohol use but, when it arose, prompted interviewees to explore the subject in detail. In line with a developmental approach to qualitative interviewing, CM developed prompts so that later interviews built on insights gleaned from earlier interviews about emergent themes [22]. Following this approach, exactly half of the women interviewed (n = 20) spontaneously mentioned alcohol during the interview. Of these, 13 women engaged in an extended dialogue with CM on the topic of alcohol use while a further seven made briefer remarks. Ten women reported having consumed alcohol during at least one of their pregnancies, while ten reported having abstained from alcohol during all of their pregnancies.

### Analysis

The motivation for this article arises from the need to add women’s perspectives to the present debate among physicians, philosophers and health researchers, as to the best way of communicating information about alcohol consumption during pregnancy. This debate is being importantly influenced by philosophical argument. Whether or not they choose to drink alcohol during pregnancy, women’s perspectives, voiced in their own terms, have an equally important role to play in validating or refuting some of the claims being made. We recognised an opportunity to add to this conversation by analysing data we had already collected. Our data reflect women's views as they arose spontaneously and were expressed candidly as our study did not frame the issue of alcohol consumption in any particular way.

An inductive ‘framework analysis’ approach was taken to coding and analysis [23] as follows: All interviews were transcribed verbatim. Data about women’s use of alcohol during pregnancy were extracted from transcripts and read by the team and key themes and issues identified after consultation among authors. The themes identified at this stage were: 1) risk; 2) sensitivity to imposed norms; 3) characteristics of self-reported drinking and 4) recalled patient-practitioner communication. These themes were then applied to the data and headings created to structure a picture of the data as a whole while preserving variation in the views expressed. In particular, we were interested in the similarities and differences in the emotional content and behaviours that were reported by women in relation to their attitudes and identities. We classified women as “drinkers” and “abstainers” respectively. These terms denote women’s behaviour during pregnancy, as self-reported, and should not to be taken as indicative of women’s drinking identities generally. Finally, interpretations and explanations based on associations in the data, initially deemed unrelated, were developed.

Our interest with this paper is in making inferences at a cultural level, relevant to understanding how ideas and values circulate in society and are consumed by individuals. To this extent, we were generally less interested in assessing whether the content of women’s self-reported behaviour was true or false and relatively more interested in the cultural implications of the views they expressed and the behaviours they talked about. In other words, we consider that the interviewer stood as a proxy for a young woman who may seek, or obtain, knowledge from the women being interviewed on the subject of alcohol consumption during pregnancy. Although we were interested in illuminating women’s experiences and decision-making about alcohol use in this article, we were careful not to ignore relevant contextual information evident in the interview data as a whole. We referred back to interview transcripts throughout analysis and incorporated contextual information into our findings where appropriate.

## Results

### Risk perceptions in relation to identity as a driver of behaviour

The interviewees predominantly shared a perspective on the risk of alcohol consumption during pregnancy, yet women followed different patterns of behaviour. Overall, women indicated that they were aware of the risk that alcohol consumption posed to the fetus but many considered that the risks of *some* alcohol consumption during pregnancy were low. As the following two representative excerpts illustrate, this commonality in risk perception was contextualised with respect to different valuations of the importance of alcohol to identity and social functioning to motivate distinct behaviours of drinking or abstinence. We define identity, here, as the qualities (including behaviours) that make someone or something what they are and different from other people. Even this simple definition helps describe why it would be important to women to continue familiar activities consonant with who they see themselves to be through a life-changing event, such as pregnancy. Two key positions were evident among the women we interviewed: 1) those for whom alcohol was not deemed an important element of one’s life and social functioning who reported easily avoiding alcohol for a “short” period of time (the term of pregnancy), and 2) those for whom drinking alcohol formed an important part of their identity and social functioning and who continued to drink or found it a burden to abstain. For example, one respondent, who in the early part of the interview explicitly stated that she thought drinking a small amount of alcohol would not have put the fetus at risk, nevertheless described that she had no motivating reason to use alcohol during pregnancy and chose to abstain:

Respondent: I just thought it was a short period of my life to not have to worry about ah, it didn’t really faze me that much, [alcohol] was just something I could easily just do without. [participant 35, abstainer]

In contrast, another woman stated:

Respondent: I continued to drink alcohol through my whole pregnancy, but only a third of a glass would be all I would have. […] I didn’t want to give [alcohol] up altogether, because it’s a bit of a social thing as well, and I enjoy the flavour of wine. I didn’t think it would hurt the kids at all, which I still don’t. [participant 12, drinker]

Here, both women professed that consuming a small amount of alcohol was a low risk activity, however, only one chose to drink. Participant 35 highlighted both “not wanting to worry” and the lack of importance she placed on consuming alcohol as reasons motivating abstinence. Participant 12 highlighted the importance of alcohol to maintaining social activities and her enjoyment of the taste of wine as factors that led her to continue to consume alcohol during pregnancy, albeit at a reduced rate. Participant 12 reported that she had given up caffeinated drinks for the duration of her pregnancies because she “didn’t think [caffeine] would be any good for a child” – indicating both her ability and willingness to sacrifice certain pleasures during pregnancy for the sake of her fetus.

Aside from making a decision about day-to-day drinking behaviour, the cultural importance of alcohol in marking special occasions and celebrations meant these were times when women either made an exception and drank or reportedly “missed” being able to consume alcohol:

Respondent: I had two glasses of wine I think with both pregnancies. Ah, I think there was a birthday and an anniversary […]. I possibly had something at Christmas, like a glass of wine at Christmas, yeah so I would’ve, maybe two maximum. [participant 18, drinker]

Respondent: So it wasn’t so hard to just kind of steer clear of [alcohol]. I would say, you know I probably would miss having a glass of wine or whatever here and there, at various [important occasions] along the way, but it wasn’t anything yeah, that ever really worried me too greatly. [participant 14, abstainer]

### Impact of perceived external judgement on maternal drinking during pregnancy

Whether they chose to drink or abstained, women’s accounts indicated that they were aware of social pressures to be a “responsible” expectant mother who abstained from alcohol. For some, this perceived external pressure was sufficient to make them abstain:

Respondent: I think some people would have looked at me a bit funny if I was having a drink. I’d you know, as I said I was a social drinker, and I didn’t [drink during pregnancy], but I think I would have copped it a bit if I did! (laughs) […] and the guilt that’s associated with it. I couldn’t well I don’t know if you could live with yourself […] you’d have to wonder if you didn’t give yourself, if you don’t give the baby the best chance and the best environment to be growing in. [participant 4, abstainer]

While Participant 4's motivators to abstain included wishing to avoid the negative judgment of others as well as the possibility of feeling “guilt” if she did not provide an ideal fetal environment, not all women described being affected by social pressures to abstain. For example, Participant 39 quoted below highlighted the uncertainty that surrounded the risk that small amounts of alcohol pose to the fetus. She thought that while some might believe that pregnant women should abstain from alcohol, this was not true of everyone:

Respondent: […] it was the food I had more concern with. Drinking, I don’t believe you know, there are certain people going, “You cannot drink at all.” But there’s a lot of people going, “Ah! I had a drink” you know? There was probably less concern knowing that I wasn’t sitting there shooting back some you know, some stiff drinks, so. [participant 39, drinker]

As with Participant 12 quoted earlier, who avoided caffeine, this woman said she was conscious of other risks such as avoiding foods associated with a risk of consuming foods associated with a risk of contracting listeria, but was less concerned about alcohol.

### Conceptualising alcohol consumption

Those who continued to drink alcohol during pregnancy described how they conceived “acceptable” levels of drinking. One glass of wine, once or twice a week, was the maximum acceptable level of drinking reported by women. Many women emphasised their reduced consumption by stating they only had “a glass of wine here or there” or by highlighting that they drank fractional glasses of wine on any given occasion. No woman said that she, knowingly, drank more than one glass on any day while pregnant.

Regardless of whether women’s reports reflected what and how much alcohol they actually consumed during pregnancy, what was notable was that women’s descriptions of what constituted acceptable drinking appeared to be based on beliefs about the perceived potency of different kinds of alcoholic beverages, and the notional unit of a glass of wine as a standard drink, rather than the alcohol content or exact volumes of the drinks they imbibed. Women in this sample spoke exclusively of their intentional alcohol consumption in terms of units of wine while depicting other forms of alcohol i.e. spirits as harmful:

Respondent: I had had a bit of a drinking session (laughs) not realising, and I then found out that I was pregnant. And it wasn’t just that one or two drinks, it was, and it was gin! I remember thinking “no I had gin!” […] I was really concerned about that fact, it was also because it would probably have been like three or four drinks, not just that odd glass of wine. [participant 18, drinker]

### Role of healthcare practitioner in maternal alcohol use during pregnancy

Overall, women’s descriptions of their experiences with their practitioners, mostly obstetricians, suggested that patient-practitioner communication about alcohol was informal and sporadic. Women tended to describe their medical practitioners as “relaxed” about their consumption of alcohol during pregnancy. One woman stated:

Respondent: I was pretty comfortable with [drinking alcohol], largely because I had both obstetricians very relaxed about alcohol, a lot more relaxed than I’d expected, and than I was. And so yeah, I’d have a glass, a couple of glasses a week, and I didn’t feel bad about it. [participant 30, drinker]

Here, it was evident that the obstetrician played a key role in helping manage women’s anxieties – their emotional state – and in some cases downplayed women’s intuition to be concerned about alcohol.

The woman quoted in the following excerpt described being conscious of not eating the foods her obstetrician advised her to avoid. She depicted her obstetrician as having thoroughly discussed the risks of eating particular foods and drinking alcohol but that he had not recommended she abstain:

Respondent: In terms of alcohol, for the first [child], I said “well what’s the go? I’m not a big drinker, I have like a glass of wine.” He said, “Well just make sure it’s a spritzer, just mix it with some mineral water.” So I do. [participant 39, drinker]

A number of women stated that they could not recall their practitioner speaking with them about alcohol consumption. While acknowledging that they may simply have forgotten, the tone of their description indicated that practitioners do not consistently discuss alcohol use in consultations with patients:

Respondent: She wasn’t someone that, and I know because I’ve had a lot of friends who’ve had babies, like some people are really pedantic about certain things. I guess I was always fairly thin, she never weighed me once, that kind of thing. […] she actually never really asked me about my eating habits. I don’t remember her asking me about my alcohol intake. [participant 30, drinker]

## Discussion

Despite Australian health guidelines advising women to abstain from drinking alcohol during pregnancy [1], as many as 80% of Australian women will drink alcohol at some point during their pregnancy [2]. While guidelines were changing when these woman were pregnant and this rate may be declining [4], this discrepancy still raises questions about how and why women make the choices they do about their alcohol consumption during pregnancy. It also raises questions as to whether, and if so how, abstinence messages should be conveyed in ways that are both ethical and effective i.e. that will not provoke anxiety.

Our findings indicate that the importance of alcohol to the social lives and identities of Australian women importantly influence their decisions whether to drink alcohol during pregnancy or not. In contrast to an earlier study, these results suggest that Australian women perceive the risk of *some* alcohol consumption during pregnancy to be low [11]. As detailed in our previous published work on this sample, the women in our study were generally risk averse [24]. This risk aversion is evidenced in the findings presented here in terms of women’s stated concerns about the risks of eating foods that could cause listeria and with imbibing caffeine. In this context, women’s lesser concern about consuming small quantities of alcohol is something of an anomaly.

The importance of alcohol to women’s social lives offers an explanation of quantitative studies showing that temporal patterns of pre-pregnancy consumption, rather than average rates, predict alcohol consumption during pregnancy [8]. Our findings also support qualitative research on Australian women’s drinking practices, more broadly, that has shown that “alcohol consumption is used in the construction and deployment of positive, highly female identities (that is, identities as women and mothers)” [25], p361.

What is remarkable is that, even from a relatively small dataset, it is clear that women can play numerous different roles in either actively perpetuating, or challenging, existing drinking norms. Those who viewed their alcohol consumption positively, and as important to them in some way, did not want to give up alcohol altogether during pregnancy. They actively advocated for their right to drink alcohol as something which was consonant with their identities as pregnant women. At the same time, however, it was evident that some women, who might otherwise have abstained, acceded to certain cultural expectations by acquiescing to celebratory drinking norms. It appeared too, that there was important variation in sensitivities among women to the different cultural values placed on women to both drink and not to drink.

The women in our study were aware of social expectations that they cease consumption of alcohol during pregnancy [26] and some responded by abstaining. Some, however, highlighted differences of opinion about the importance of abstinence. Those who continued to drink during pregnancy reported feeling comfortable with their decision, albeit while being careful to articulate that they drank at a reduced rate according to a self-defined set of rules (one or two glasses of wine per week and no more than one on any occasion). Desire to avoid “worry” or “guilt” were given as sufficient reasons to abstain except where their practitioner (mostly an obstetrician) was deemed to have moderated these emotions by supporting their alcohol consumption.

This complexity makes it difficult to provide any “one size fits all” guidance to practitioners in having conversations with pregnant women about alcohol consumption. This is so because practitioners potentially need to adapt their advice based on their patient’s sensitivity and where she sits regarding the different cultural norms at play. If we were to classify women in a binary fashion according to three variables: alcohol and identity (important, not important), knowledge of risk (risky, not risky) and sensitivity (sensitive to judgement, not sensitive to judgement), there could be as many as eight distinct profiles to engage. While some women may benefit from simple advice as to the risks of consuming alcohol, others may benefit from self-esteem building or practical strategies as to how they might confidently refuse alcoholic beverages in social situations if they do not wish to drink. In highlighting cultural pressures at play, however, we show that this issue is not solely the responsibility of a pregnant woman and her practitioner. Targeting the social-environment, by advocating for women’s (and men’s, as well) right to refuse to drink without question i.e. creating abstinence supportive social-environments, is equally important.

We found that some women expressed anxiety about drinking alcohol during pregnancy but this anxiety was not widespread. Women most often identified their sources of anxiety as a perceived societal disapproval of drinking rather than discouragement of drinking by their obstetricians, who were generally depicted as “relaxed” about using alcohol during pregnancy. Women indicated they were careful to heed their obstetricians’ advice regarding food. We found no evidence that women contemplated abortion due to anxiety over alcohol consumption and also no evidence that obstetricians played any role in amplifying anxiety about women’s alcohol consumption [15–17, 19]. The women’s accounts clearly conveyed that they viewed their obstetricians’ advice as authoritative, and were motivated to comply with their recommendations. The accounts show that obstetricians are highly influential in helping women to manage guilt and anxiety in relation to their alcohol consumption during pregnancy.

The way women reported their alcohol consumption, typically in terms of number of glasses of wine, offers insight into the way women conceptualise the alcohol they are drinking. Specifically, it suggests one of two possibilities about what wine symbolises to women: 1) that it is a more “healthy” alcoholic beverage than others, a view consonant with the common media discourse as to the health benefits of wine [27]. It is possible that wine, in contrast to beer or spirits may be viewed as a less harmful, or even beneficial, kind of alcoholic drink and/or 2) that it has a strong symbolic association with femininity, the use of which reinforces positive female identities [25]. Furthermore, talking about alcohol in relation to the idea of a standard drink, and a kind of alcoholic beverage, indicates that women are not thinking about the ethanol content they consumed but rather in terms of a more generic measure of volume. Finally, women’s discussion of alcohol consumption was in terms of its social importance, and in one case taste. Discussion of alcohol’s toxicology, biological or physiological effects were completely absent in their discourse, the closest such reference being the one woman quoted who described her body as being an “environment” for her baby to grow in. This social rather than chemical conceptualisation of alcohol has potential implications for how women deliberate over the risk of conditions like Fetal Alcohol Spectrum Disorder (FASD), an assessment of which requires one to think about alcohol as a teratogen.

## Conclusion

We present these qualitative findings to shed light on the reasons why Australian women consume alcohol or not during pregnancy and the impact abstinence messages may have on their drinking activities and broader sense of wellbeing during pregnancy. Our findings show the extent to which drinking during pregnancy places women between competing cultural norms that simultaneously encourage drinking, through the ubiquity of alcohol use on social occasions, and discourage drinking, through the disapproval of drinking during pregnancy and health guidelines that promote abstinence. Risk was an important consideration for women, however, concern with risk was clearly offset by awareness of the disputed evidence of the harm alcohol poses the fetus and socially and identity based discourse that informed decisions to drink or avoid alcohol. Few women described experiencing anxiety around their drinking practices. For those who did this was a powerful motive to abstain. Women’s doctors, who were not generally described as advocating abstinence, were important mediators of women’s drinking practices and the emotional dimensions of this. We found no evidence that practitioners provoked anxiety by engaging in conservations with women about the risks of alcohol consumption during pregnancy.

The key finding here is that women’s drinking behaviours during pregnancy are embedded in their broader cultural contexts and the emotionally laden engagements women have with others, including healthcare professionals, in their social environment. Although further quantitative research is needed to establish the generalizability of these results, our findings suggest two provisional implications for policy and practice: 1) Public health messages to assist in curtailing drinking during pregnancy may be enhanced by a) communicating strategies to enable women to more effectively decline to drink alcohol, if they do not want to; b) disseminating messages to the broader population as to why they should not make women feel compelled to drink alcohol; c) providing information that wine is not necessarily a more “healthy” alcoholic drink than others; and d) communicating to women about alcohol as ethanol – a substance that is a teratogen; 2) healthcare professionals should realise the potential power they have in managing women’s anxieties about alcohol consumption, and be aware of the implications of the different types of perspectives women can have in tailoring their advice, but need not be concerned about raising anxiety by engaging in a respectful dialogue with women about the risks of alcohol consumption during pregnancy.

### Limitations and future research directions

One limitation of this study is that it does not present findings on women who drank alcohol during pregnancy but did not admit doing so to the interviewer. This would likely include those most sensitive to negative external judgements on women’s drinking during pregnancy but who nevertheless drank alcohol during pregnancy, potentially at high levels. This group is likely to be particularly vulnerable and difficult to engage. We have some anecdotal evidence to suggest that the open-ended approach our study took may have led to greater disclosure by women of behaviours deemed socially undesirable, such as drinking alcohol while pregnant, than qualitative studies where alcohol consumption during pregnancy is the explicit study aim. Understanding and engaging women who drink during pregnancy but are strongly disposed to conceal their consumption, is an important area of study which will require passive methods of observation and/or the use of qualitative research methods that protect their anonymity e.g. web-based studies.

As women acknowledged themselves, recall bias is likely. However, we do not believe such a bias affects the validity of our findings, because we were mostly interested in making inferences about the character of women’s talk, rather than whether the content was true or false, and because our aim was to represent an exhaustive range of views rather than to offer estimates of prevalence of any particular view. Our findings are relevant to the experiences of White Australian women of higher socioeconomic status. They were in an older age group during their last pregnancy and most could afford to receive private maternity care and to consult an obstetrician. However, this is also a group of women more disposed to consuming alcohol during pregnancy [4, 21].

The women in this study were pregnant at the cusp of a change to guidelines from guidelines recommending low alcohol consumption to guidelines recommending abstinence. As such, our analysis is aimed, simply, to illuminate the perspectives of these women regarding abstinence messages. It does not offer an analysis of the “impact” of abstinence guidelines as would require a before-after quasi-experimental design. However, if we assume, as seems likely, that beliefs and behaviour change will at best lag the publication of official guidelines, these women can be seen to represent a cohort influenced by low alcohol consumption guidelines. As a result, we can view these data as a benchmark for assessing changes in qualitative responses following the change in guidelines. An interesting avenue for future research would be to carry out a similar study with a sample of women who have a similar profile as this group, in order to examine any trends in beliefs and behaviours over time.

Further research is needed into how women from other age groups, socioeconomic groups and ethnicities make decisions about their alcohol consumption during pregnancy. We also need to investigate obstetricians’ and other healthcare professionals’ level of concern about alcohol consumption during pregnancy, how they vary their advice according to women’s socioeconomic status and ethnicity, and what their particular approaches are to dialogues about alcohol use. Further investigation of different communication strategies appropriate for women, differentiated in terms of the role of alcohol in identity, knowledge of risk and sensitivity, could be helpful to practitioners.
